# Annual Meeting West Texas Medical Society; East Line Medical Society: The East Texas Medical Society

**Published:** 1887-12

**Authors:** 


### Society Notes.

### ANNUAL MEETING AND BANQUET OF THE WEST TEXAS MEDICAL ASSOCIATION.

A polite note from the secretary of this thriving association, Dr. Fred Terrell, informs us that on the 30th of October the West Texas Medical Association held its annual meeting in San Antonio, and indulged their annual custom of winding up with a sumptuous banquet. Dr. F. Herff was elected president, and Drs. P. W. Johns and Edward Bennett vice-presidents, while Dr. Fred Terrell was re-elected secretary and treasurer. The new board of censors consists of Drs. Braunagle, A. Herff and J. V. Spring. The following members participated in the banquet at the Menger Hotel, and speeches were made and toasts given and responded to over choicest wines. till a late hour: President Herff, Drs. Geo. Cupples, F. Terrell, G. G. Watts, A. Herff, E. Bennett, D. Berry, B. F. Kingsley, Amos Graves, J. Braunagle, S. T. Lowery, L. L. Shropshire, C. E. R. King, E. Herzberg, Ed Carothers, P. W. Johns, and last, but not least, Drs. T. J. Tyner and B. E. Hadra, residing now in Austin.

We are glad to note the continued growth and prosperity of this old and stable society.

THE EAST LINE MEDICAL SOCIETY meets in Sulphur Springs first Tuesday in February. Dr. Paine, of Comanche, will be present by invitation, and will lecture on electricity in medicine, and demonstrate his "electrical diagnosis". A large attendance is expected.

THE EAST TEXAS MEDICAL ASSOCIATION will meet next at Jacksonville, on Tuesday morning, January 3, 1888. Regular physicians are solicited to join. Fifty members now. No fees, fines or dues exacted. Leading object, medical discussions and professional intercourse. Original papers and reports solicited. All interested are welcome to attend.

J. E. MAYFIELD,

NACOGDOCHES, November 26, 1887. Secretary.

